# Conservation of Vital Force

**Published:** 1878-01

**Authors:** J. Williams


### CONSERVATION OF VITAL FORCE.

BY DR. J. WILLIAMS.

*Upon retiring from the Presidency of the Ohio State Dentai Society, December 7th, 1877.*

*Gentlemen of the Ohio Dental Association:*

The fields of thought most appropriate to an occasion like the present, have been fully explored by my predecessors.

Dental education, literature, societies, jurisprudence, ethics, etc., have been duly considered, in these annual addresses. I ask your pardon, therefore, for the presentation of some thoughts that many seen somewhat foreign to the peculiar class of subjects and questions that call us together in these annual convocations. Trusting, however, that you will consider the subject that I shall present, not wholly inappropriate.

How our lives may be prolonged, in good working order, to the greatest possible extent, and how the highest degree of physical strength and mental vigor may be attained and conserved, are, it has seemed to me, questions worthy of our most careful consideration.

There are times when we feel abounding life and energy, every nerve readily conveying the vital fluid to the muscles called into action by the will. At such times we feel master of the situation, whatever it may be; to work or think is a pleasure, a positive luxury. To arrive at a knowledge of the laws on which such conditions depend, and how such physico mental states may be made more generally to obtain, may well engage the attention of thinking and working men in any profession or vocation in life. All may not acquire perfect physical organizations; all have not been endowed with a possibility so desirable; but all can economize the protoplasm of life and develope the greatest possible amount of *vital force* in the organizations that have been transmitted to them. Often have persons, possessing very feeble constitutions, by a careful husbanding of all their forces, accomplished more, suffered as little, and lived longer, than others who have been splendidly endowed. The robust are often prodigal of their abundance and speedily become bankrupt.

The intellectual faculties may have acquired the broadest and deepest culture, such culture as may be obtained by a thorough mastery of the branches usually embraced in the best college curriculum, supplemented by a thorough course of training under private preceptors, and in the medical and dental schools. There may exist the finest moral and social qualities; the hand and fingers may have acquired a marvelous amount of skill and cunning; all this culture, let us hope, the COMING dentist will have; but what would all these splendid acquirements of "head, heart and hand," be without an adequate amount of vital force? Like the grand and beautiful locomotive with the steam left out, like a ship without a sail.

Where is the compensation for the time, money and energy expended in the acquisition of all this literary and professional culture and training, if *superannuated*, or *deceased* is to be written over it all, before life's sun has reached the meridian. Too many splendid wrecks mark the pathway of professional life. It is as much the duty of professional men to point out the causes that inevitably lead to physical and mental enervation, as to impart a knowledge of the principles on which the successful practice of any of the learned professions is based. The long continued hard work of life can only be accomplished well by those having full vital force and an iron will. Resistance can only be overcome by an equal amount of force.

The highest, most perfect and spontaneous action of the mental faculties, as memory, judgment, original suggestion, etc. depends on a full supply of life force. The physician or dentist, weary and exhausted with over work, must necessarily fail in diagnosis, and the application of proper remedial agents. The mind of the surgeon perceives conditions and relations, and decides upon a course proper to be pursued; and the bodily organs execute the plans and purposes of the judgment. The execution will be perfect or defective according to the bodily condition. Why does the hand of one individual, in the performance of a delicate operation, tremble like the aspen leaf, while every nerve and muscle of another, not superior to the first in culture and skill, is firm and steady, perfectly executing the purposes of the judgment and will? The first approached his work destitute of that full supply of force which only can give firmness and precision. The second was thoroughly equipped with strength and self-control. Vital force thoroughly under control of the judgment and will, gives confidence, to both patient and operator; while a manifest want of it, *discourages* the operator, and *disgusts* the patient. How often has the weak, timid patient been stimlated and encouraged by the cool, deliberate, kind dentist or surgeon.

The most elaborate research has been made by scientific men to discover the primal origin and inception of life, and its resultant, vital force. Whether life has its origin in a spiritual entity in conjunction with organization, or whether it results from organization alone, are question on which scientific men are divided; and which they are not likely soon to settle. What phenomena may attend the existence of spirit disconnected with, and independent of organization, is beyond the province of science to determine. This much science has demonstrated; life has a physical basis; and without materiality, there is no manifestation of life or vital force. When the living human organism receives within itself, food of a proper character, it is subjected to numerous changes and modifications; and there is evolved a something that we call vital force, or energy; certain phenomena that are called mental, and others that are called physical. Whether these phenomena exist *solely* on account of a certain molecular arrangement of the particles of matter, derived from the food, which have become part of the living organism, we certainly are not able to determine. One thing only we do know: without the food, none of these phenomena could have any existence, discernable by any of our senses.

It is a fact then, verified constantly, that food is an essential factor in the production of vital phenomena. To say that it is the *one efficient factor*, would be to assume more than can be demonstrated or verified by any known method of induction or experiment. It is enough for our present purpose to know that it is an essential factor; and that the kind of food has something to do in the degree of force evolved. These are knowable things, and of much practical importance.

Material substances cannot be indiscriminately appropriated by the assimilative functions. So far as I am able to ascertain, it is not in the power of the animal organism to appropriate and convert *mineral compounds* into *living tissue.* The order of nature is, that plants in their growth, appropriate the mineral compounds, and so change them, that these same elements may be appropriated by the living animal organism, and become part of it. And again, after having been elevated from the vegatable to the animal plain, these same elements may become food for other animal organizations, be readily appropriated; and in their metamorphosis produce the constant phenomena of *force.*

It seems to me most natural to infer, and it accords with facts well established, that after vegetable protoplasm has been elevated by the inferior animals to that plane, it is then, as it exists in the healthy inferior animal, best suited to become food, in a large degree, of human beings, and its conversion into our own tissue is effected with less loss of force than most other kinds of food. True, those of the vegetarian persuasion claim exactly the opposite; but their theory seems lacking in matter of fact demonstration. Those animals that live on the flesh of other animals, are as a rule, distinguished for their strength, activity and sagacity. "The lion is the king of beasts." Certainly it cannot be shown that physical and mental force finds its highest and most perfect exemplification in those men who have entirely discarded animal food as an article of diet. We believe that the best authorities in the world are agreed upon this; that good fresh beef, mutton, wild game, fowls and fish, contain the greatest amount of nutriment, in the most convenient form. I speak of the diet of healthy persons only. The diet of diseased persons must be considered from a pathological stand point.

Prof. Agassiz, says: "The fish enters largely into the requisition of the human system. It is a kind of food which refreshes the system, especially after intellectual fatigue. There is no other article of food that supplies the waste of the head so thoroughly as fish diet; and the evidence of it is in the fact that all the inhabitants of the sea shores, the world over, are the brighter population of the country. Fish contains phosphorus to a large extent; a chemical element which the brain requires for growth and health. We would not say that an exclusive use of fish, would make a blockhead a wise man, but that the brain should not be wanting in, or of its essential elements."

Ex-Governor Seymore says, "That there is more nutritious food in an acre of water, well stocked with fish, than in the best wheat growing farm in New York." Let each one test this matter for himself; and he will know of the doctrine whether it be true or false.

The superiority of the well to do farmer over the poorly fed and poorly clad common laborer, is very largely owing to the fact, that the former eats more and better food than the latter. The superiority of the officer over the common soldier has a similar explanation.

There can be no thought, no volition, no action without food; and by *increasing* the quantity and *improving* the quality, our ability to work either mentally or physically, will be increased. The law manifests itself in this, that when action is increased, the demand for food is increased.

The influence of sun-light on animal and vegetable life, has been observed by all, and yet thousands, who might otherwise be strong and healthful, are weak and feeble, because they are deprived to a great extent of its health giving influence. All must admit, whether able to understand the chemical effects of sun-light or not, that without it, all vegetable and animal life would soon become extinct. There is not a flower, plant or tree, that can perfect itself if grown in the shade; and there is no animal life developed into healthful activity and energy, when deprived habitually of sun light. Unfortunately the rays of the sun produce a shade of color not generally admired; but better sun-browned cheek, than the pale delicacy of weakness. Sun-light is natures great energizing element; and those who enjoy most of it, other things being equal, are the most energetic, the most healthful. It may be questioned whether those rooms into which the sun's rays never enter, are fit to work in, to sleep in, or even to be sick in. Every bone, muscle and nerve, every vital organ, every tissue is influenced for healthy growth and activity, by a plentiful supply of the pure light of day.

Dr. Franklin's great discovery, that sun light is cheaper than candle light, is full of blessing to those who are wise enough to take the hint. Many noble men in our profession are weak, and die prematurely, because they do not use, as they should, this great panacea of nature.

Pure air, in its effects upon the system, is closely allied to sun light. It seems to me; that no intelligent man, in his sober senses, could allow himself to be packed, in a crowded audience, for an hour or more, in a badly ventilated room. If it were possible to *see* the impurity floating in the air of crowded audiences, with but little provision for admitting pure air, and no provision for the escape of the impure, every man, woman and child would escape as precipitately as if the building were on fire.

To say nothing of the many cases of speedy death from breathing badly vitiated air, it is a well ascertained fact, that to breathe only moderately impure air, day after day, for a considerable period of time, is productive of very serious consequences. The teachers in our public schools are usually sufferers from this cause; as their palid faces, partial loss of appetite, decline in vivacity and decrease of muscular strength well attest. Bronchitis and phthisis pulmonalis, frequently result from the same cause.

Not only should the laws by which force is generated be well understood, but the means by which it may be conserved, that it may be applied "where it will do the most good," should receive attention likewise.

Some men earn an abundance of money, but never have any when they need it, because they spend it without thought or consideration. Some men spend their vital force in the same manner. No man can have a good supply of energy on hand, who is constantly making drafts upon it, without allowing sufficient time for rest and recreation to intervene. Some men seem to entertain the idea that spending a week or two, once a year, at some fashionable watering place, where all the laws of health are usually violated, is about all that is needed in the way of rest, diversion and exercise. This will not meet the demands of the tired mental and physical organs. If there be daily *wear*, there must be daily *recuperation*.

Horse back riding furnishes by far, the greatest amount of invigoration, in the shortest period of time. We have by this means, the exercise and increased circulation, with but little loss of force, because very little voluntary effort is required.

Action either of the mind or body necessarily uses up force; and the more complexity, difficulty, uncertainty, risk or responsibility of the work to be done, the greater will be the loss of force. These occupations that make large drafts on the physico mental faculties are the most exhausting; and, except in the most powerful constitutions, such occupations can only be pursued through a long life time, by wisely and judiciously allowing periods of rest and diversion to follow work.

The premature death of that most eminent Surgeon, Dr. Metz, of Massillon, O., is a sad example of over-work, in professional life.

Nothing so effectually prevents an excessive loss of vital force in the performance of delicate operation, as conscious ability to accomplish the work in a thorough and skillful manner. Where doubts and uncertainty exist, there will be worry; and worry is always more exhausting than work. Hence the importance of young men, designing to enter the dental profession, or any other profession, laying the foundation broad and deep in thorough general and special culture, before assuming the responsibilities of professional life.

In conclusion, gentlemen of the Ohio Dental Association, allow me to thank you, *most sincerely*, for the honor you have conferred in calling me to preside over your deliberations.

—*Transactions Ohio State Dental Society.*

